# Primary Vitreoretinal Lymphoma: A Retrospective Study of 20 Eyes

**DOI:** 10.1155/2022/4522974

**Published:** 2022-07-01

**Authors:** Jing-Yi Luo, Shi-Tong Yu, Xiao-Yu Xu, Xian-Xuan Lin, Rong-Jiang Luo, Chong-De Long

**Affiliations:** ^1^State Key Laboratory of Ophthalmology, Zhongshan Ophthalmic Center, Sun Yat-Sen University, Guangdong Provincial Key Laboratory of Ophthalmology and Visual Science, Guangdong Provincial Clinical Research Center for Ocular Diseases, Guangzhou, Guangdong 510060, China; ^2^Department of Ophthalmology, The First Affiliated Hospital, Sun Yat-Sen University, Guangzhou, Guangdong 510080, China; ^3^Department of General Surgery & Guangdong Provincial Key Laboratory of Precision Medicine for Gastrointestinal Tumor, Nanfang Hospital, The First School of Clinical Medicine, Southern Medical University, Guangzhou, Guangdong 510150, China; ^4^C-MER Guangzhou Dennis Lam Eye Hospital, Guangzhou, Guangdong 510030, China

## Abstract

**Purpose:**

This study aimed to describe and analyze the clinical features of 20 eyes of 15 primary vitreoretinal lymphoma (PVRL) patients.

**Methods:**

This was a retrospective case series and a review of the literature. Fifteen PVRL patients (20 affected eyes) referred between February 2011 and December 2019 were recruited, and their medical records were retrospectively reviewed.

**Results:**

Among these 15 PVRL patients, seven were men (46.67%), and five had bilateral PVRL (33.33%). The median onset age was 66 ± 9.26 years and six (40%) patients had central nervous system (CNS) involvement, and two of them died of CNS-related complications. The ocular symptoms varied from decreased vision to binocular diplopia. The ocular manifestations were diverse and involved both the anterior and posterior segments, including the vitreous cells, subretinal white-yellow lesions, cotton-wool spots, and ophthalmoplegia. The rate of misdiagnosis and failure to diagnose was 100%, and 30% of them were misdiagnosed as uveitis. We found five cases revealing rare characteristics of this malignancy. Among them, there were two cases with mild hypertensive retinopathy exhibiting cotton-wool spots, one case mimicking age-related macular degeneration (AMD), one case with systemic lupus erythematosus (SLE), and one patient had extraocular muscle involvement. To the best of our knowledge, we reported PVRL exhibiting cotton-wool spots as the main manifestation and coexisting with extraocular myopathy for the first time.

**Conclusions:**

PVRL is a rare intraocular malignancy that commonly masquerades as uveitis. As the clinical signs and symptoms are atypical, ophthalmologists must carefully examine patients to avoid misdiagnosis or a failure to diagnose. Cotton-wool spots and extraocular myopathy might be the dominant initial symptoms in PVRL patients, and AMD should be considered a differential diagnosis of PVRL. SLE patients under immunosuppressive treatment could have spontaneous PVRL.

## 1. Introduction

Primary vitreoretinal lymphoma (PVRL) is a rare malignancy that occurs in the retina or vitreous of the eye, comprising less than 0.01% of all ocular diseases [1, 2]. PVRL most commonly represents non-Hodgkin's lymphoma (NHL), and 90% of cases have diffuse large B-cell lymphoma (DLBCL) [3]. The clinical features of PVRL are nonspecific, including the presence of large, nonclumped vitreous cells and white-yellow subretinal deposits associated with solid retinal pigment epithelium (RPE) detachments [4], making it one of the most common masquerade syndromes [5, 6]. The differential diagnosis of PVRL is wide and includes infectious and noninfectious uveitis, retinal vasculitis, white dot syndrome, metastatic cancers, and so on [7–12]. The delay in diagnosis of PVRL varies from 4 to 40 months [8, 13, 14], and up to 90% of PVRL cases develop central nervous system (CNS) lymphomas during disease progression, resulting in a poor prognosis and a low survival rate [1, 15]. Therefore, establishing an early diagnosis of PVRL allows for early treatment, which may be beneficial to reducing the mortality rate [16].

In the current study, we described and analyzed the clinical features of 15 PVRL patients to extend the existing knowledge of this ocular malignant tumor, hoping to facilitate an earlier diagnosis by ophthalmologists.

## 2. Methods

This is a retrospective case series. Written informed consent was acquired from all of the patients or their next of kin. The study adhered to the tenets of the Declaration of Helsinki and obtained Institutional Review Board approval from Zhongshan Ophthalmic Center, Sun Yat-sen University (IRB number: IIT2022057).

Patients with biopsy-proven PVRL from Zhongshan Ophthalmic Center, Sun Yat-sen University (ZOC), and the First Affiliated Hospital of Sun Yat-sen University (FAHSYSU) between February 2011 and December 2019 were included. The diagnosis of PVRL was based on a positive ocular tissue biopsy by vitrectomy or vitreous aspirate. For cases in the absence of ocular tissue biopsy, the diagnosis depended on a positive extraocular tissue biopsy by bone marrow aspirate or lumbar puncture. The clinical specimens were sent to senior pathologists to assess the pathological features by morphological assessment, immunocytochemistry, or molecular techniques. Exclusion criteria included ocular and head trauma, ocular surgery history, extraocular lymphoma history, systemic infectious diseases, acquired immune deficiency syndrome, and other immunodeficiency diseases.

Medical records of the PVRL patients, including initial symptoms, medical history, best-corrected visual acuity (BCVA), IOP with a Goldmann applanation tonometer, slit-lamp biomicroscopy, ophthalmoscopy, fundus photography (CR-2 AF, Digital Retinal Camera, Canon, Japan), optical coherence tomography (OCT, Spectralis OCT, Heidelberg Engineering, Germany), and magnetic resonance imaging (MRI), were searched in the case management systems of ZOC and FAHSYSU or obtained from the patients themselves. Coexisting systemic syndromes were evaluated by experienced oncologists or neurologists at FAHSYSU at the time of diagnosis. Patients were followed by the ophthalmologist and their medical oncologist every six to 12 months, and the data regarding chemotherapy and radiotherapy were also collected. Cases with special characteristics are described in detail. A statistical description was generated using SPSS for Windows, version 26.0.

## 3. Results

Fifteen patients (20 affected eyes) with biopsy-proven PVRL were recruited for our study, including seven men (8 eyes) and eight women (12 eyes). Five patients (33.33%) had bilateral PVRL at presentation. The median age at diagnosis was 66 ± 9.26 years (range, 49–81 years) with a median delay in diagnosis of 5 ± 4.35 months (range, 0–14 months). The median follow-up time was 28.5 ± 10.60 months (range, 12–45 months). During follow-up, six patients (40%) subsequently developed CNS lymphoma, and two of them died of CNS-related complications. Neither acquired immunodeficiency syndrome nor other immunodeficiency diseases were found in these patients.

All 15 patients had varying degrees of ocular signs as their first symptoms, and the most common signs were decreased vision (9 patients, 11 eyes) and floaters (7 patients, 9 eyes). Other symptoms included photophobia in four patients (5 eyes), ocular pain in three patients (3 eyes), and binocular diplopia in one patient (1 eye). Six patients (40%) had constitutional symptoms such as fever, headache, hypomnesia, and aphasia (Table 1). On the ocular examination, the BCVA at the patients' first visit varied from light perception to 20/25. The median baseline IOP was 14 ± 7.69 mmHg (range, 8–38 mmHg), and four eyes (20%) had elevated IOP.

**Figure 1:**
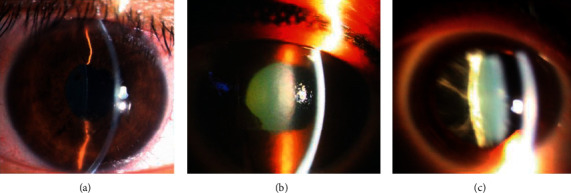
Anterior segment photography of primary vitreoretinal lymphoma (PVRL) patients. (a) The right eye of a 52-year-old female patient with PVRL shows moderate conjunctival and episcleral congestion, pigmentary keratic precipitates, mild aqueous flare, and iris posterior synechiae, mimicking iridocyclitis. (b) The right eye of a 49-year-old female PVRL patient demonstrates severe corneal edema, mild aqueous flare, iris posterior synechiae, and cataract. She complained of mild eye irritation when she came to our clinic. (c) A 69-year-old male PVRL patient exhibits mild conjunctival and episcleral congestion, moderate cataract, and severe vitreous opacity with massive floaters in his right eye.

Clinical signs in both the anterior and posterior segments of the eyes were observed in all PVRL patients (Table 2). The characteristics of the anterior segment included conjunctival and episcleral congestion (5 eyes), corneal edema (2 eyes), keratic precipitates (KP) (6 eyes), aqueous flare (7 eyes), iris posterior synechiae (2 eyes), rubeosis iridis (1 eye), sluggish light reflex (4 eyes), and asymmetric cataract (1 eye). The Tyndall signs of aqueous flare were mild to moderate with a few aqueous humor cells (Figures 1(a) and 1(b)).

**Figure 2:**
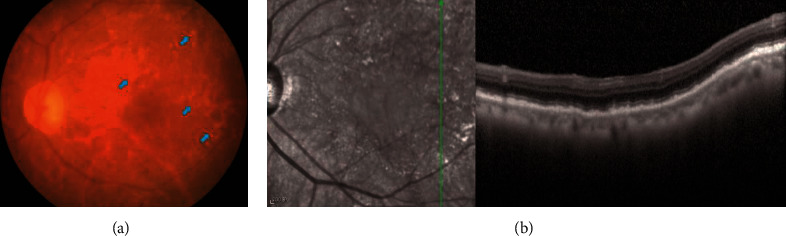
Fundus examination of the left eye of a 67-year-old male patient with primary vitreoretinal lymphoma. (a) Color fundus photography reveals RPE irregularities and subretinal white-yellow lesions with varied sizes and numbers in the posterior pole of the left eye (blue arrows). (b) The white-yellow lesions exhibit sub-RPE hyperreflective deposits on optical coherence tomography.

**Figure 3:**
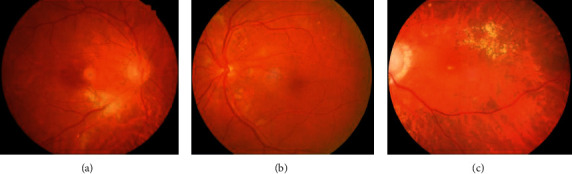
Color fundus photography of primary vitreoretinal lymphoma (PVRL) patients. (a) A 67-year-old male hypertension patient with PVRL presents with large integrated cotton-wool spots, with linear hemorrhagic lesions and punctate yellow-white spots in his right fundus. The retinal arteriolar is attenuated. The patient was misdiagnosed with hypertensive retinopathy. (b) A 52-year-old female PVRL patient shows Purtscher-like retinopathy. Color fundus photography of the left eye reveals multiple confluent cotton-wool spots around the optic nerve head and along the temporal arcades. Superficial dot hemorrhages, microaneurysms, and tiny yellow-white lesions are also visible in the posterior pole. (c) A 48-year-old PVRL male patient has multifocal yellow-white lesions and hemorrhagic loci in the macula of his left eye, resembling age-related macular degeneration.

The posterior segment abnormalities of the PVRL eyes had variant clinical features. All 20 affected eyes exhibited varying degrees of vitreous opacity (Figure 1(c)). Seventeen of them underwent retinal examinations, while three eyes with severe cataracts or vitreous opacities could not be examined. All these 17 eyes exhibited vitreous cells and RPE irregularities in the fundus. Punctate subretinal white-yellow lesions were found in 12 eyes, which showed different sizes with obscured boundaries and predominantly clustered in the posterior poles (Figure 2). Some white-yellow foci were easily missed by ophthalmoscopy observation (Figure 3(a)). Retinal hemorrhages were present in 5 eyes. Two eyes from two different patients exhibited cotton-wool spots (Figures 3(b) and 3(c)). Other manifestations included optic nerve edema (4 eyes) or atrophy (2 eyes), macular edema (3 eyes), hard exudates (3 eyes), sub-RPE infiltration lesions (3 eyes), retinal vascular leakages (2 eyes), retinal neovascularization (1 eye), and exudative retinal detachment (1 eye). Ophthalmoplegia, which is a periorbital sign, was also found in one patient in our study.

Before biopsy confirmation of PVRL, the patients were diagnosed with various masquerading conditions (Table 2). The most common misdiagnosis was uveitis in six eyes (30%). Other diagnoses included conjunctivitis, scleritis, vitritis, age-related macular degeneration (AMD), infectious endophthalmitis, retinal vasculitis, neovascular glaucoma, hypertensive retinopathy, Purtscher's retinopathy, and Tolosa–Hunt Syndrome. Three patients were referred for an unknown disease. Nearly half of the patients (7 cases) had neurological symptoms at approximately nine months (range, 7–18 months) after developing ocular conditions. Cerebral lesion(s) were found in these patients and they were finally diagnosed with DLBCLs by pathological examination. Individuals in our study received an array of treatments before being diagnosed with PVRL, including steroids in eight patients, topical in eight, and oral in four. Other recorded therapies were topical antibiotics, topical glaucoma drops, nonsteroidal anti-inflammatory, vitrectomy, and intravitreal injection of anti-vascular endothelial growth factor (anti-VEGF). Three patients received no treatment before diagnosis.

Five specific cases require further elaboration. Three patients suffered from systemic diseases, including systemic hypertension in two patients and systemic lupus erythematosus (SLE) in one patient. When they came to the ophthalmic clinic, their ocular abnormalities were first considered to be retinopathy associated with these chronic systemic diseases. For the two patients with hypertension, their fundus exhibited cotton-wool spots and microhemorrhagic foci that mimicked hypertensive retinopathy (Figure 3(a)) and Purtscher-like retinopathy (Figure 3(b)), respectively. However, as both patients had vitreous floaters indicating additional inflammatory signs, vitreous aspirates were performed at their follow-up visits and the diagnosis of DLBCL was established. For the patient with SLE, she had visual acuity deteriorating and her fundus examination showed retinal vasculitis and tiny subretinal white-yellow lesions. Punctate white-yellow loci were also found in these patients, but the lesions were tiny and easily missed under ophthalmoscopy examination. She was at an inactive stage of SLE and was currently under low-dose maintenance treatment with immunosuppressants and glucocorticoids. The final diagnosis of non-Hodgkin's lymphoma was established by vitreous biopsy. After local and systemic methotrexate chemotherapy, the number and size of cotton-wool spots and white-yellow lesions decreased. The fourth case was a 48-year-old male who had central vision loss for six months. His fundus examination revealed mild vitreous opacity and macular hard exudates with hemorrhagic lesions (Figure 3(c)). However, the hemorrhagic lesions were minimal under funduscopy and were neglected by the clinical doctor at the first visit. After exclusion of systemic and infectious diseases, he was diagnosed with dry AMD. The lesions had obviously expanded after a 3-month follow-up. Cytokine analysis of vitreous fluid found elevated IL-10 and IL-6 at 436.8 pg/ml and 93.6 pg/ml, respectively. A diagnostic vitrectomy was performed and the cytology and immunohistochemistry studies confirmed the diagnosis of DLBCL. Subsequent MRI of the brain and orbits found no intracranial or orbital space-occupying lesions. Another case was a 60-year-old woman. She complained of fever, binocular diplopia, and pain in the right eye and orbit for ten days after her first visit to the ophthalmic clinic. The BCVA was 20/40 in the right eye and 20/25 in the left eye. Ocular examination found mild lens opacity in both eyes and a few vitreous cells and exotropia position with limited movement toward the nasal direction in the right eye. An MRI showed a mildly enlarged right cavernous sinus. The primary diagnoses were established as cataract, vitritis, and Tolosa–Hunt syndrome. The patient was referred to the neurology clinic for further examination, and a cerebrospinal fluid test confirmed the diagnosis of NHL. After chemotherapy with rituximab and methotrexate, her symptoms gradually improved and the number of vitreous cells decreased. However, the patient died of neurological complications within two years.

## 4. Discussion

In the current study, we investigated the clinical features of 20 eyes of 15 patients with PVRL to gain a better understanding of this disease, hoping to improve the early diagnosis rate and prolong survival as much as possible. We reported five cases revealing rare characteristics of this malignancy. Among them, two cases with mild hypertensive retinopathy exhibited cotton-wool spots, one case mimicked AMD, one case had SLE, and one patient had extraocular muscle involvement. To the best of our knowledge, we reported PVRL exhibiting cotton-wool spots as the main manifestation and coexisting with extraocular myopathy for the first time.

PVRL, a subset of primary central nervous system lymphoma (PCNSL), [9] is a rare intraocular lymphoid malignancy, and its atypical clinical features make early diagnosis difficult [17]. As the symptoms of PVRL vary from decreased vision to binocular diplopia and ocular lesions involving both the anterior and posterior segments, the rate of misdiagnosis and failure to diagnose is high [12, 18, 19]. Mortality is up to 81% in PVRL patients with CNS involvement [20, 21]. Early systemic treatment for PVRL might delay the onset of CNS lymphomas and improve the prognosis [22].

In our study, 53.33% of the patients were men, and the median onset age was 66 years, and 40% of patients had CNS involvement, which is consistent with previous reports [8, 13]. However, only 33.33% of patients presented with binocular retinal lymphomas, and this lower proportion compared with previous studies [2, 23] might be related to the limited sample size and relatively short observation time. Although HIV and Epstein–Barr virus infection are important risk factors for PVRL, [9, 24, 25], none of our patients were infected.

The symptoms of 15 PVRL patients varied from decreased vision to binocular diplopia, and the ocular disorders involved both the anterior and posterior segments. The rate of misdiagnosis and failure to diagnose was 100% with an average delay in the final diagnosis of 5 months. Given the nonspecific symptoms and clinical signs that mimic ocular inflammatory conditions, most of the cases were misdiagnosed as uveitis (6/20 eyes, 30%), which is similar to previous studies [10, 18, 19]. As PVRL is the most common type of masquerade syndrome, it is challenging and vital to establish a correct diagnosis. Their nonspecific clinical features also share common features with other ocular diseases.

Cotton-wool spots were prominent fundus findings in two cases in our study. Hyun et al. reported a case with rapidly developing cotton-wool spots in both eyes as an initial sign of systemic NHL [26]. Retinal cotton-wool spots are considered a significant clue to systemic diseases, and they might be produced by a focal ischemic insult [27]. In PVRL pathogenesis, microinfarction by tumor emboli and immune-related vasculitis may contribute to this microvascular change. Nonetheless, cotton-wool spots are features of moderate hypertensive retinopathy [28]. For the differential diagnosis, hypertensive retinopathy should present the same or more severe degree of accompanying microangiopathy, such as arteriovenous nicking, hard exudates, and blot or flame hemorrhages, not consistent with our cases [29, 30]. Moreover, both cases had vitreous floaters indicating additional inflammatory signs. Then, vitreous aspirates were performed at their follow-up visits and the diagnosis of DLBCL was established.

Both PVRL and AMD exhibit invasion of the RPE and sub-RPE or subretinal deposits, making them have similar clinical manifestations [31]. The yellow-white sub-RPE or subretinal deposits at the macula in the early stage of PVRL resemble drusen in AMD. However, in PVRL cases, the lesion may become enlarged and confluent in a short period, and some patients have complaints of floaters because of vitreous cells. Sub-RPE vertical hyperreflective deposits on OCT may also be helpful in identifying diseases [3, 32]. Our patient previously diagnosed with Tolosa–Hunt syndrome is another misdiagnosed PVRL case. Tolosa–Hunt syndrome, characterized by recurrent painful ophthalmoplegia caused by granulomatous inflammation of the cavernous sinus region, is a diagnosis of exclusion [33, 34]. The case in our study had clinical features similar to Tolosa–Hunt syndrome, including unilateral orbital pain, paresis of the third cranial nerve, and cavernous sinus, which resulted in an incorrect diagnosis. Ocular adnexal involvement might result from lymphoma infiltration into the superior orbital fissure and was coincident with the vitreoretinopathy, suggesting a special subtype of PCNSL. When middle-aged and elderly individuals suffer from painful ophthalmoplegia with simultaneous intraocular manifestations and constitutional symptoms, careful examinations and follow-ups are required to exclude other causes of disease.

For patients suffering from cardiovascular, endocrine, or autoimmune diseases, clinicians are inclined to establish an ocular diagnosis associated with these systemic disorders. There has been one case of PVRL with SLE reported in the previous literature [35]. SLE is a multisystemic autoimmune disease involving various organs and tissues. The incidence of retinopathy in SLE is between 3% and 29% and is related to disease activity [36]. Clinical findings of SLE retinopathy include cotton-wool spots, microaneurysms, dot hemorrhages, and hard exudate [37, 38]. Otherwise, SLE patients have a higher incidence of malignancy and are four times more likely to develop NHL than healthy people [39]. The possible explanations for the association between NHL and SLE include elevated cytokines, usage of immunosuppressive drugs, and functional defects of suppressor T-cells in SLE patients [35, 39]. The SLE case in our study had deteriorated visual acuity and retinopathy progression during an inactive stage of SLE, indicating that other pathogeneses, such as malignancy, may be involved.

The limitations of our study include its retrospective nature, the small number of patients given the rarity of this disease, and a lack of detailed clinical and pathological data for some patients.

## 5. Conclusions

Since PVRL is a rare and life-threatening disease, rapidly establishing the correct diagnosis is crucial to improving patients' visual outcomes and survival. However, with its diversity and nonspecificity of clinical manifestations, a diagnosis can be challenging. Detailed and systemic examination as well as follow-up observation help in the early diagnosis of PVRL. The present study reveals that cotton-wool spots and extraocular myopathy might be the dominant initial symptoms in PVRL patients, and AMD should be considered a differential diagnosis of PVRL. SLE patients under immunosuppressive treatment could develop spontaneous PVRL.

## Figures and Tables

**Table 1:** Symptomatology of 15 PVRL patients with 20 affected eyes.

Ocular symptoms^*∗*^	Patients (%)	Eyes (%)
Decreased vision	9 (60)	11 (55)
Floaters	7 (46.67)	9 (45)
Photophobia	4 (26.67)	5 (25)
Ocular pain	3 (20)	3 (15)
Binocular diplopia	1 (6.67)	1 (5)
Constitutional symptoms		
Fever	1 (6.67)	
Headache	2 (13.33)	
Hypomnesia	2 (13.33)	
Aphasia	1 (6.67)	

^*∗*^Eight patients (53.33%) had more than 2 symptoms. PVRL, primary vitreoretinal lymphoma. Eight patients (53.33%) had more than 2 symptoms. PVRL, primary vitreoretinal lymphoma.

**Table 2:** Clinical characteristics and prior diagnosis of 20 eyes with PVRL.

Clinical characteristics (>1 possible)	Number (%)
Anterior segment	*n* = 20
Conjunctival and episcleral congestion	5 (25)
Corneal edema	2 (10)
Keratic precipitates (KP)	6 (30)
Aqueous flare	7 (35)
Iris posterior synechiae	2 (10)
Rubeosis iridis	1 (5)
Sluggish light reflex	4 (20)
Asymmetric cataract	1 (5)
Posterior segment^*∗*^	*n* = 17
Vitreous cells	17 (100)
RPE irregularities	17 (100)
Subretinal white-yellow lesions	12 (70.59)
Retinal hemorrhages	5 (29.41)
Cotton-wool spots	2 (11.76)
Optic nerve edema	4 (23.53)
Optic nerve atrophy	2 (11.76)
Macular edema	3 (17.65)
Hard exudates	3 (17.65)
Sub-RPE infiltration	3 (17.65)
Retinal vascular leakages	2 (11.76)
Retinal neovascularization	1 (5.89)
Exudative retinal detachment	1 (5.89)
Periorbital	*n* = 1
Ophthalmoplegia	1 (100)
Prior diagnosis (>1 possible)	*n* = 20
Conjunctivitis	2 (10)
Scleritis	1 (5)
Uveitis	6 (30)
Vitritis	2 (10)
AMD	1 (5)
Infectious endophthalmitis	1 (5)
Retinal vasculitis	1 (5)
Neovascular glaucoma	1 (5)
Hypertensive retinopathy	2 (10)
Purtscher's retinopathy	1 (5)
Tolosa–Hunt syndrome	1 (5)
Uncertain	3 (15)

^*∗*^Retinal examinations were performed in 17 eyes. PVRL, primary vitreoretinal lymphoma; AMD, age-related macular degeneration.

## Data Availability

The detailed data used to support the findings of this study are available from the corresponding author upon request.

## References

[1] Mochizuki M., Singh A. D. (2009). Epidemiology and clinical features of intraocular lymphoma. *Ocular Immunology and Inflammation*.

[2] Chan C.-C., Rubenstein J. L., Coupland S. E. (2011). Primary vitreoretinal lymphoma: a report from an international primary central nervous system lymphoma collaborative group symposium. *The Oncologist*.

[3] Deák G. G., Goldstein D. A., Zhou M., Fawzi A. A., Jampol L. M. (2019). Vertical hyperreflective lesions on optical coherence tomography in vitreoretinal lymphoma. *JAMA Ophthalmology*.

[4] Llorenç V., Fuster C., Alba-Linero C. (2019). Clinical features of primary and systemic metastatic intraocular lymphomas in Spanish patients. *Journal of Ophthalmology*.

[5] Karma A., Von Willebrand E. O., Tommila P. V., Paetau A. E., Oskala P. S., Immonen I. J. (2007). Primary intraocular lymphoma. *Ophthalmology*.

[6] Coupland S. E., Heimann H., Bechrakis N. E. (2004). Primary intraocular lymphoma: a review of the clinical, histopathological and molecular biological features. *Graefe’s Archive for Clinical and Experimental Ophthalmology*.

[7] Chawla R., Venkatesh P., Garg S. P., Mandal S., Tewari H. K. (2005). Cytomegalovirus retinitis in a patient with non-hodgkin’s lymphoma: a diagnostic dilemma. *European Journal of Ophthalmology*.

[8] Cassoux N., Merle-Beral H., Leblond V. (2000). Ocular and central nervous system lymphoma: clinical features and diagnosis. *Ocular Immunology and Inflammation*.

[9] Sagoo M. S., Mehta H., Swampillai A. J. (2014). Primary intraocular lymphoma. *Survey of Ophthalmology*.

[10] Browning D. J., Fraser C. M. (2007). Primary intraocular lymphoma mimicking multifocal choroiditis and panuveitis. *Eye*.

[11] Shah G. K., Kleiner R. C., Augsburger J. J., Gill M. K., Jampol L. M. (2001). Primary intraocular lymphoma seen with transient white fundus lesions simulating the multiple evanescent white dot syndrome. *Archives of Ophthalmology*.

[12] Gill M. K., Jampol L. M. (2001). Variations in the presentation of primary intraocular lymphoma. *Survey of Ophthalmology*.

[13] Hoffman P. M., McKelvie P., Hall A. J., Stawell R. J., Santamaria J. D. (2003). Intraocular lymphoma: a series of 14 patients with clinicopathological features and treatment outcomes. *Eye*.

[14] Grimm S. A., Pulido J. S., Jahnke K. (2007). Primary intraocular lymphoma: an international primary central nervous system lymphoma collaborative group report. *Annals of Oncology*.

[15] Davis J. L. (2013). Intraocular lymphoma: a clinical perspective. *Eye*.

[16] Hormigo A., Abrey L., Heinemann M.-H., DeAngelis L. M. (2004). Ocular presentation of primary central nervous system lymphoma: diagnosis and treatment. *British Journal of Haematology*.

[17] Hoog J., Dik W. A., Lu L. (2019). Combined cellular and soluble mediator analysis for improved diagnosis of vitreoretinal lymphoma. *Acta Ophthalmologica*.

[18] Takase H., Arai A., Iwasaki Y. (2022). Challenges in the diagnosis and management of vitreoretinal lymphoma—clinical and basic approaches. *Progress in Retinal and Eye Research*.

[19] Kase S., Namba K., Iwata D. (2021). Clinical features of primary vitreoretinal lymphoma: a single-center study. *Cancer Diagnosis & Prognosis*.

[20] Venkatesh R., Bavaharan B., Mahendradas P., Yadav N. K. (2019). Primary vitreoretinal lymphoma: prevalence, impact, and management challenges. *Clinical Ophthalmology*.

[21] Akpek E. K., Ahmed I., Hochberg F. H. (1999). Intraocular-central nervous system lymphoma. *Ophthalmology*.

[22] Klimova A., Heissigerova J., Rihova E. (2018). Combined treatment of primary vitreoretinal lymphomas significantly prolongs the time to first relapse. *British Journal of Ophthalmology*.

[23] Levasseur S. D., Wittenberg L. A., White V. A. (2013). Vitreoretinal lymphoma. *JAMA Ophthalmology*.

[24] Steffen J., Coupland S. E., Smith J. R. (2021). Primary vitreoretinal lymphoma in HIV infection. *Ocular Immunology and Inflammation*.

[25] Cochereau I., Hannouche D., Geoffray C., Toublanc M., Hoang-Xuan T. (1996). Ocular involvement in epstein-barr virus-associated T-cell lymphoma. *American Journal of Ophthalmology*.

[26] Hyun S. C., Yoon Y. H. (2003). Rapidly developing cotton-wool spots as the first manifestaion of systemic non-hodgkin’s lymphoma. *Retina*.

[27] Brown G. C., Brown M. M., Hiller T., Fischer D., Benson W. E., Magargal L. E. (1985). Cotton-wool spots. *Retina*.

[28] Tan W., Yao X., Le T.-T., Tan B., Schmetterer L., Chua J. (2022). The new era of retinal imaging in hypertensive patients. *Asia-Pacific Journal of Ophthalmology*.

[29] Wegmann-Burns M., Gugger M., Goldblum D. (2004). Hypertensive retinopathy. *The Lancet*.

[30] Keith N. M., Wagener H. P., Barker N. W. (1974). Some different types of essential hypertension: their course and prognosis. *The American Journal of the Medical Sciences*.

[31] Riazi-Esfahani H., Hassanpoor N., Ghassemi F., Zarei M. (2020). Presumed intraocular lymphoma masquerading as age-related macular degeneration: a case report. *Journal of Current Ophthalmology*.

[32] Barry R. J., Tasiopoulou A., Murray P. I. (2018). Characteristic optical coherence tomography findings in patients with primary vitreoretinal lymphoma: a novel aid to early diagnosis. *British Journal of Ophthalmology*.

[33] Jarholm J. A., Faiz K. W., Nysted T., Zarnovicky S., Kristoffersen E. S. (2018). Orbital pain, ophthalmoplegia, and oligoclonal bands in the cerebrospinal fluid: a case report of tolosa-hunt syndrome. *Headache: The Journal of Head and Face Pain*.

[34] Dutta P., Anand K. (2021). Tolosa-hunt syndrome: a review of diagnostic criteria and unresolved issues. *Journal of Current Ophthalmology*.

[35] Woei-A-Jin F. J. S. H., Kersting S., Bollemeijer J. G. (2012). Primary intraocular lymphoma in a patient with systemic lupus erythematosus. *Annals of Hematology*.

[36] Dammacco R. (2018). Systemic lupus erythematosus and ocular involvement: an overview. *Clinical and Experimental Medicine*.

[37] Palejwala N. V., Walia H. S., Yeh S. (2012). Ocular manifestations of systemic lupus erythematosus: a review of the literature. *Autoimmune Diseases*.

[38] Alzahrani A. S., Alqahtani W., Hazzazi M. A., Alqahtani A. S. (2022). The application of optical coherence tomography angiography in a patient with systemic lupus erythematosus. *Cureus*.

[39] Bernatsky S., Boivin J. F., Joseph L. (2005). An international cohort study of cancer in systemic lupus erythematosus. *Arthritis & Rheumatism*.

